# Changes in granulosa cells gene expression associated with growth, plateau and atretic phases in medium bovine follicles

**DOI:** 10.1186/1757-2215-7-50

**Published:** 2014-05-07

**Authors:** Gabriel Douville, Marc-André Sirard

**Affiliations:** 1 Centre de Recherche en Biologie de la Reproduction, Faculté des Sciences de l’Agriculture et de l’Alimentation, Département des Sciences Animales, Pavillon INAF, Université Laval, Québec G1V 0A6, QC, Canada

**Keywords:** Granulosa cells, Transcriptome, Growth status, Cow

## Abstract

**Background:**

The objective of this study was to build the transcriptomic profile of granulosa cells originating from follicles 6 to 9 mm in diameter in dairy cattle using microarrays.

**Methods:**

Granulosa cells originating from three different phases of antral follicle growth were compared: growing (G), plateau (P) and atresia (A), as categorized by flow cytometry profiles of DNA. The growing and atretic conditions were each hybridized against the plateau condition as a reference in order to understand the specific biological mechanisms modulated in this class of follicles.

**Results:**

2,942 genes were differentially expressed (P < 0.05) in P vs. G and 1,974 in A vs. P. A clear segregation of the 3 phases was confirmed by between group analysis (BGA). The first characteristic of the plateau phase is the activation of the upstream regulators *TP53* and *PTEN* which participate in the reduction of cell growth through *MYC*, *FOS* and *E2F1-2-3.* We also observed the down-regulation of steroidogenesis genes: *CYP11A1* and *CYP19A1*, in the granulosa cells of the plateau phase relative to the growth phase. On the other hand, the A vs. P contrast showed up-regulation of multiple transcripts associated to apoptosis: *CCT2, DAB2, DSG2* and *TGM2*.

**Conclusions:**

This study offers multiple candidate genes to be further studied in order to elucidate their role in the modulation of follicular development and, ultimately, of oocyte quality.

## Background

At birth, female mammals have a reserve of primordial follicles, tallied at around 130,000 in the cow [1]. Primordial follicles consist of an immature oocyte, arrested at prophase I of meiosis, surrounded by a single layer of somatic, granulosa cells (GC) [2]. By a mechanism that has yet to be well defined [3], a new cohort of these primordial follicles is constantly being activated to start their development, which progresses through a primary stage, a secondary stage and after around 140 days, in cows, eventually reach the antral stage. The antrum is a fluid-filled cavity that forms within the follicle when the latter has reached a diameter of around 250 μm and its oocyte is around 90 μm in diameter [4]. Unlike in mice [5] where antrum formation corresponds to the oocyte achieving meiotic competence, the ability to continue meiosis past the diplotene phase of prophase I, cattle oocytes only acquire this competence once the follicle reaches a diameter of about 3 mm, which corresponds to an oocyte diameter between 110–120 μm [6]. A transient rise in FSH serum levels will initiate a follicular growth wave, an event referred to as: recruitment [7]. The FSH levels will peak and start decreasing when the biggest follicles from the recruited cohort reach 4-5 mm in size [8]. This decrease in FSH will eventually lead to deviation, where the leading follicle will continue its growth and become dominant while the growth rate of subordinate follicles will be reduced and they will eventually regress by atresia [9]. On average, deviation occurs 2.5 days after emergence of the largest follicle of the wave, the latter reaching a diameter of 8.5 mm, while the largest subordinate in the wave is around 7.7 mm [8]. With the exception of the dominant follicle, follicles are destined for atresia, the former having acquired a relative independence from FSH [10] characterised by features such as being able to respond to LH signal by expressing functional LH receptors in its granulosa cells [11].

The objective of this study was to analyze the transcriptomic profile of follicles in the FSH-dependent growth phase (G) relative to follicles in the plateau phase (P) as a result of FSH deprivation, and of plateau follicles relative to follicles in the advance atresia stage (A). Using flow cytometry to measure DNA content of GC originating from individual follicles, the precise follicular growth stage was assessed in order to investigate genes pathways and families associated with these physiological states.

Pathway analysis software was used to study the biology underlying each phenotype.

## Methods

All chemicals were obtained from Sigma-Aldrich (Oakville, On, Canada) unless otherwise stated.

### Granulosa cells collection

Slaughterhouse ovaries from dairy cattle were kept on ice during transport and throughout the collection procedure in order to maintain RNA integrity. Individual follicle (6-9 mm) collection of the mural granulosa cell layers were recovered by scraping and measures were taken to avoid contamination between samples [12,13]. Briefly, the follicle diameter was measured with a ruler at the ovary surface and part of the follicular fluid was aspirated using an 18 gauge syringe in order to facilitate scraping. The hole made by the needle was widened by cutting out a triangular piece of ovarian epithelium using dissecting scissors. A small, rounded weighing spatula with no abrasive surface was then inserted through the hole and used to gently scrape the follicular walls, limiting the chances of collecting theca cells. Although we cannot rule out minor thecal contamination, this procedure is different form follicle aspiration with a needle where large chunk of theca cells can contaminate one sample. Therefore if contamination would be generated, it would be likely similar in all samples, and therefore would not impact gene expression ratios.

Granulosa cells from each follicule were transferred to individual 1.5 ml centrifuge tube containing 500 μl PBS solution without Ca^2+^ or Mg^2+^ (pH of 7.1 recipe (g/L): 0.2 KCL, 0.2 KH_2_PO_4_, 8.0 NaCl, 1.14 Na_2_HPO_4_, 1.0 D-glucose (dextrose), 0.11 pyruvate (pyruvic acid), passed through 0.22 μm filter) containing 125 mg/L EDTA (PBSE) [14]. Tubes were kept on ice prior to and after receiving the granulosa cells, again to maintain RNA integrity. In order to avoid contamination between samples, the instruments were rinsed in distilled water, dipped in 70% ethanol solution and wiped before collecting from the next follicle.

The samples were passed through a light vortex (half speed for 5 seconds) and centrifuged at 600 × g for 30 seconds at 4°C. The supernatant was discarded to remove cellular debris or blood in the sample leaving about 10 μl without disturbing the pellet, especially for small follicles where it was barely visible. Immediately after supernatant removal, the pellet was re-suspended by light vortexing in 50 μl of the same cold PBSE solution described above.

For each sample, the 50 μl cell suspension, 25 μl was transferred to a new 750 μl tube on ice for propidium iodide (PI) staining and flow cytometry analysis. The volume remaining in the original 1.5 ml tubes was frozen for RNA extraction.

### Flow cytometry

The following procedures were done in cold solutions to avoid lysing the cells. For each sample, 250 μl cold PBSE solutions (kept on ice) were added to the 25 μl suspension assigned to cytometry (also kept on ice). 340 μl of cold 95% EtOH was slowly added to the cell suspension, while vortexing at half-speed, to attain a final concentration of 70%. The samples were fixed in this alcohol suspension for a minimum of 16 h at 4°C. This permeabilized the granulosa’s membrane, allowing the DNA fragments resulting from apoptosis to exit the cell, giving a lower DNA content detectable by flow cytometry [14,15].

The staining procedure was adapted from [14]. Briefly, the fixed samples were centrifuged at 600 × g for 2 min at room temperature to remove the alcohol and DNA fragments. The granulosa pellet was then re-suspended in 500 μl of PI solution (50 μg/ml) in PBSE containing 0.1% Triton X-100, and RNase A at 50 μg/ml (50 U/mg) [16]. The samples were then incubated 30 min at room temperature, allowing enough time for the RNase to eliminate RNA, leaving only DNA as stained content within the cells.

The flow cytometry to analyze the DNA content in PI-stained cells was performed with a Beckman-Coulter EPICS XL (Mississauga, On) equipped with a 488 nm laser. Forward light scatter (FSC) is an indicator of relative particle size and side light scatter is an indicator of granularity. Both were used to gate out debris during the acquisition process. Peak versus integrated fluorescence were used to gate out doublets; two cells stuck together. Integrated fluorescence essentially indicates the time a particle took to pass the laser for a given event, two cells stuck together taking longer. 10,000 events were acquired once the gates had been applied.

The fluorescence histograms resulting from flow cytometry were modelized using the FlowJo software. The modelization gave the proportion of total events in the sub-G1 phase (G1-), corresponding to cells with less than 2n DNA content, G1 phase, corresponding to cells containing 2n of DNA, S phase, corresponding to cells that are duplicating their DNA material, G2 phase, corresponding to cells having completely duplicated their genetic material to 4n.

The FlowJo software was limited as the model it applied contains approximations and the numbers are rounded [17]. Consequently, the sum of the proportions of phases did not add up to exactly 100%, but varied between 92.83% and 110.54% for the samples collected in this experiment. The proportions were normalized by dividing the proportion of a given phase by the sum of all proportions, and multiplying the result by 100.

To categorize the samples from 6–9 mm follicles, they were first ordered in decreasing order using the following formula:

$$G2+S+G1-\left(G1-\right)=x$$

26 samples were collected from 26 individual follicles in the 6–9 mm diameter category. Only one follicule per ovary was selected. Based on the formula above, the samples were separated in 3 groups: The 8 samples with the highest x value were categorized as the "growing" (G) group, the 8 samples with the lowest x values were categorized as the "atretic" (A) group and the 8 samples with intermediate x values (few mitosis and limited atresia) were categorized as the "plateau" (P) group, while the 2 samples remaining at the boundaries were not included in the rest of the analysis.

### Sample selection for microarray analysis

4 samples were selected per category for micro array analysis. The quality of the RNA was tested on a Bioanalyser and any sample with a RIN value lower than 8.5 was discarded, excluding the advanced atretic follicles. Within the growing follicles, the 6 samples with the highest G2 + S : G1 ratio were considered and within these 6, the 4 samples with the highest G2 + S : G1- ratios were selected for hybridization. To narrow down to samples showing a "treadmilling" characteristic, the 6 samples with a G2 + S : G1- ratio nearest 1 in the plateau follicle group were considered, and among them, the 4 samples with the highest proportion of cell in the G0/G1 phase were selected for hybridization. Out of the 4 plateau granulosa samples selected, one sample was lost during the RNA extraction process and had to be replaced by the sample with the next highest G0/G1 proportion. This was the only case where a substitution had to be made. Finally, the 6 atretic follicles with the lowest G1: G1- ratio were considered, and among them, the 4 samples with the lowest G2 + S : G1 ratio were selected for hybridization, obviously favouring a relatively higher proportion population of cells undergoing apoptosis, corresponding to overall atresia for the follicle.

### Microarray

Total RNA extraction was performed using *PicoPure RNA* Isolation kit, (Life Technologies) and including a DNase digestion (Qiagen, Toronto, On) step. RNA quality was verified with 2100 Bioanalyzer (Agilent, Mississauga, On), using RNA 6000 Nano reagents, and all hybridized samples had an RNA integrity number (RIN) between 8.5 and 10.0. Using 5 ng of extracted total RNA as starting material, the linear amplification of the mRNA fraction was performed using RiboAmp HS^Plus^ RNA Amplification Kit (Life Technologies) which uses T7 RNA polymerase *in vitro* transcription (IVT) to yield antisense RNA (aRNA). The four samples from each condition were then labelled with either Cy3 or Cy5 dyes using the ULS Fluorescent Labelling Kit for Agilent arrays (Kreatech, Amsterdam, The Netherlands). 825 ng of labelled samples were prepared for hybridization using a Gene Expression Hybridization Kit (Agilent), step during which the Agilent spike was incorporated. The prepared samples were hybridized onto the Agilent-manufactured EmbryoGENE bovine microarray slide [18]. The 4 selected granulosa samples originating from individual follicles in the growing category were hybridized individually on each of the 4 arrays on the slide, against the 4 selected plateau samples, giving 4 biological replicates for each condition. Those same 4 plateau samples were also hybridized on a second slide, this time against the atretic follicles. For each contrast, a second slide was hybridized, inversing the color assigned to each condition, in order to produce a dye-swap, technical replicate. Hybridization was performed using the automated HS Pro 400 hybridization station (Tecan, Männedorf, Switzerland). The automated process consisted of: a wash using Prehybridization Buffer (Agilent) at 65°C, injection 65 μl of sample, hybridization at 65°C for 17 h, wash with GE Wash Buffer 1 (Agilent) at room temperature, wash with GE Wash Buffer 2 (Agilent) at 37°C and finally a drying step of 2 min at 30°C. The microarray slides were read using Tecan PowerScanner with the Autogain procedure on each individual array. The images were then processed with Array-Pro Analyzer 6.3.1 (Media Cybernetics, Rockville, MD) to map each spot and to manually exclude from the analysis spots obstructed by debris, such as dust particles. Expression data was then inputted into microarray analysis software FlexArray 1.6.1 [19]. Background correction was not performed to avoid a fold-change effect resulting only from low level expression following background subtraction [20]. Raw data normalization within arrays was performed using the Loess algorithm. Normalization between arrays was done using Quantile normalization, which uses intensities. The data were deposited into the Gene Expression Omnibus: GSE56145. Genes to be investigated based on their differential expression were selected based on a symmetrical raw fold change of >1.5 and a p-value of <0.05. The contrasts were set up in the following way: P vs. G and A vs. P, where a positive fold change indicated up-regulation in P and A, respectively (see Additional file 1 and Additional file 2).

**Figure 1:**
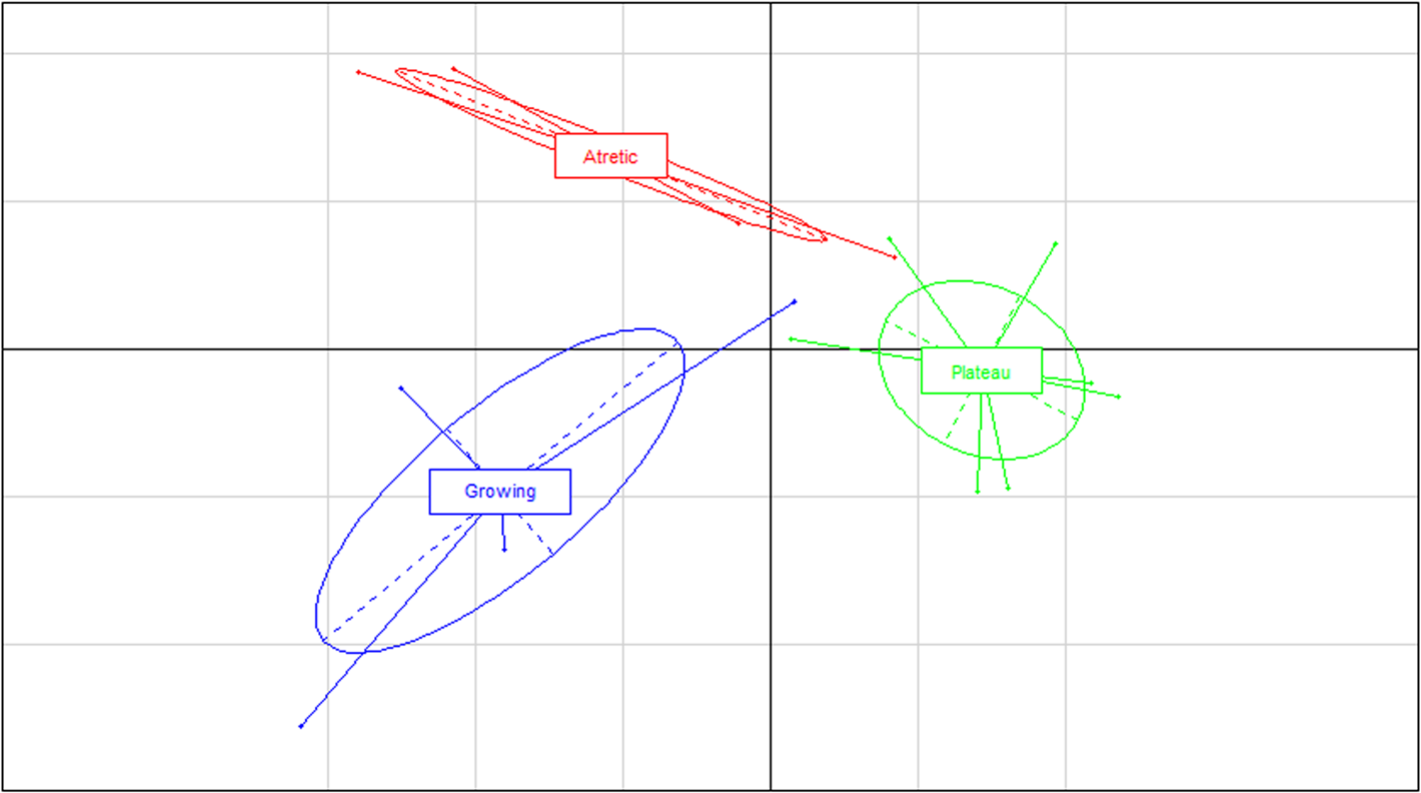
**A between group analysis (BGA).** The analysis was performed using R software [22] and using all probes’ data from the 3 folliculogenesis phases: Growing (Blue), Plateau (green) and Atretic (red).

A between group analysis (BGA) [21] using R software [22] was performed in order to make a preliminary assessment of the efficiency of cytometry to separate the GC samples in relation to their follicular origin (Figure 1).

### Analysis of differentially-expressed genes

Ingenuity Pathway Analysis (IPA) software was used to further identify functional attributes of the differentially expressed transcripts and to establish the interactions existing between the differentially expressed genes within the dataset and with other molecules in the IPA database to clarify what is driving their transcription. The limma data for all 38,732 probes on the EmbryoGENE bovine array targeting a transcript for both contrasts: P vs. G and A vs. P, were uploaded as individual dataset into the IPA software. A "core analysis" was performed separately for each using default parameters. The cut-off values were set to >1.5 and <0.05 for the fold change and p-value respectively. As an added feature not available in DAVID, for example, the results of Functions component of the IPA core analysis not only allowed transcripts to be grouped under a particular functional annotations, but it was also used to determine which functions were likely increased or decreased by integrating the direction of the fold change of a particular molecule and its documented impact on that function in the literature. IPA calculates a z-score for each functional category where a positive z-score predicts that the biological process is trending towards an increase, and a negative z-score corresponds to a decreasing trend. Z-scores ≥ 2 or ≤ -2 indicates that the function’s trend is statistically significant. The p-value, calculated by Fisher Exact Test, measures the likelihood that the genes in a dataset could be randomly associated to a given function. P-values < 0.05 (-log(p-value) > 1.3) were considered statistically significant, non-random associations. Level 1 – high-level functional category (e.g. cancer), Level 2 – mid-level category (e.g. growth) and Level 3 – specific functions, significantly represented in the data (e.g. growth of tumor), were described in the Functions component of the analysis and relevant annotations were selected among those for further investigation. Information at the Level 2 mid-level category was taken under consideration; however the p-value and z-score from the Level 3 – specific functions of the same name, encompassing all the genes within the Level-2 category, were used for the analysis.

The Transcription Factors component of the core analysis was used to determine which transcription factors (TF) were likely activated or inhibited and to select candidates downstream of those TF whose activity was relevant in the respective phase. The My Pathway tool was used to construct gene interaction network in a schematic form.

### Real-time PCR

Gene expression profiles were validated using real-time PCR (qRT-PCR). The method used to categorize and select follicles for hybridization was repeated to include an additional 3 samples in the validation. This resulted in a total of 7 distinct biological replicates per category, with the exception of the plateau phase. In the latter, only 2 additional samples were available due to a loss of samples during the extraction process or a RIN <7.0, which resulted in a total of 6 samples. Therefore, for each category, the qRT-PCR validation was performed with the 4 samples selected for hybridization and the next 3 samples, or 2 samples for plateau, best characterizing the given growth phase, as determined by cytometry. qRT-PCR was performed with non-amplified material. Total extracted RNA was reverse transcribed with oligo-dT primer and qScript Flex cDNA Synthesis Kit (Quanta Biosciences, Gaithersburg, MD). Primers specific to the targeted transcript were designed using PrimerQuest (Integrated DNA technologies**,** Coralville, IA). The qRT-PCR analysis was performed using LightCycler 480 SYBR Green I Master and the LightCycler 480 System (Roche, Laval, Qc, Cdn). The agarose gel-purified PCR product was diluted from 1 ng × 10^-4^ to 1 ng × 10^-8^ to calculate a five point standard curve in order to quantify the reaction output. Data normalization was performed based on GeNORM normalization [23] using actin, beta (*ACTB*), beta-2-microglobulin (*B2M*) and glyceraldehyde-3-phosphate dehydrogenase (*GAPDH*) as reference genes. Purified PCR products were sequenced to confirm specificity. Primer sequences, product size, annealing temperature, and accession numbers are available in Additional file 3.

GraphPad Prism 5.02 (GraphPad Software Inc.) was used to assess significant differences between genes’ relative qRT-PCR expression. Initially, a parametric one-way analysis of variance (ANOVA) was performed with a Newman-Keuls post–hoc test at a significance level alpha = 0.05, and a post-hoc test for linear trend at significance level alpha = 0.05, to determine if the slope between the expression mean of each follicular phase, G, P and A, for a given gene, was significantly greater or lower than zero.

For genes where Bartlett test for equal variance found the difference between variances to be highly significant (P < 0.001) between the three folliculogenesis phases, the data was transformed using the formula Y = Log(Y *1e12), where multiplying by a large factor was meant to obtain values higher than 1, as the log of a proportion between 0 and 1 would give a negative value, and simply multiplying by -1 would not respect the original relative expressions.

## Results

### Cytometry

**Figure 2:**
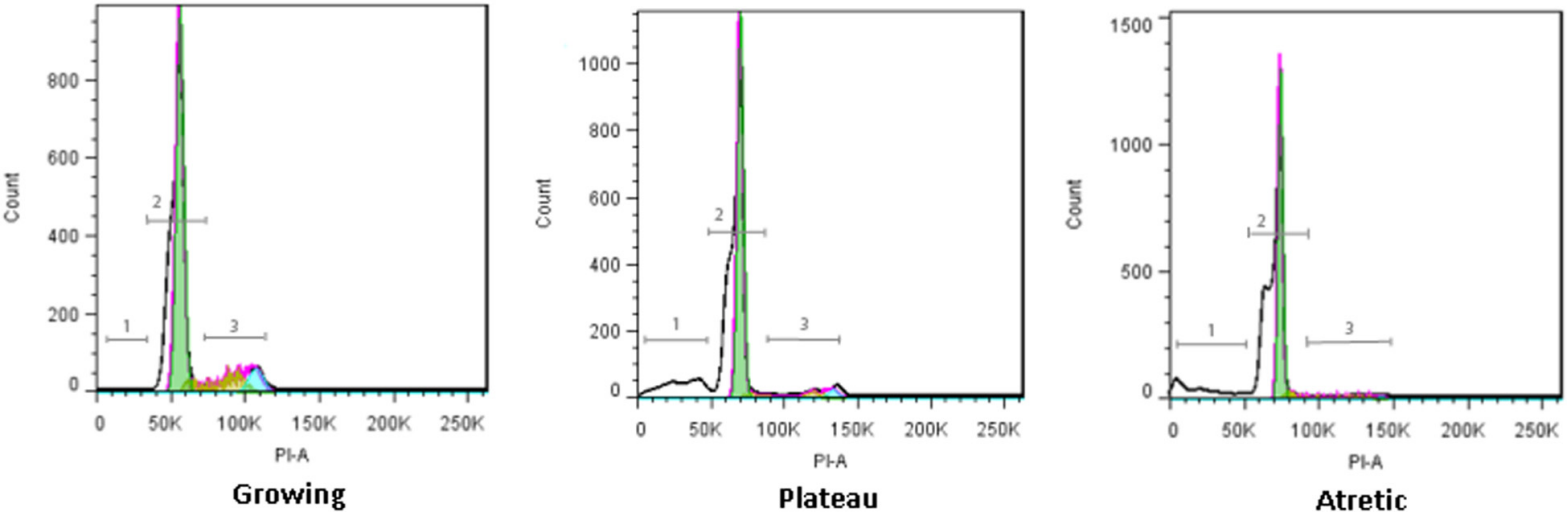
**Histogram of modeled DNA content for follicles 6-9 mm in diameter at different growing phases.** The modelization of the growing, plateau and atretic phases, using FlowJo software, gave the proportion of total events in the sub-G1 phase (-G1), corresponding to cells with less than 2n DNA content, G1 phase, corresponding to cells containing 2n of DNA, S phase, corresponding to cells that are duplicating their DNA material, G2 phase, corresponding to cells having completely duplicated their genetic material to 4n. (n = 26 granulosa samples). On the graph, the selected regions correspond to 1) –G1, 2) G0/G1, 3) S.

For the selected samples to be hybridized on the microarray, the histograms resulting from flow cytometry measurement had the expected profile for each of the respective phases based on previous investigation [14] (Figure 2). Granulosa cells from growing follicles showed a clear population in the G2 phase, corresponding to a peak to the right of the G0/G1 peak, with an intermediate S population and very few atretic cells in the shoulder along the left of the G0/G1 peak. Plateau phase follicles gave a granulosa cell population with reduced but still visible proportion G2 and S phase events, while the proportion of apoptotic cells was markedly increased as seen by a broad peak well to the left of the G0/G1, corresponding to a well-advanced stage of DNA fragmentation within the cells’ nucleus [14]. Finally, the atretic follicle profiles showed literally no cells in the S or G2 phase, an even narrower G0/G1 peak and a considerable proportion of cells in apoptosis, with a population having shifted to the very left of the fluorescent intensity axis.

### Differentially expressed genes

**Figure 3:**
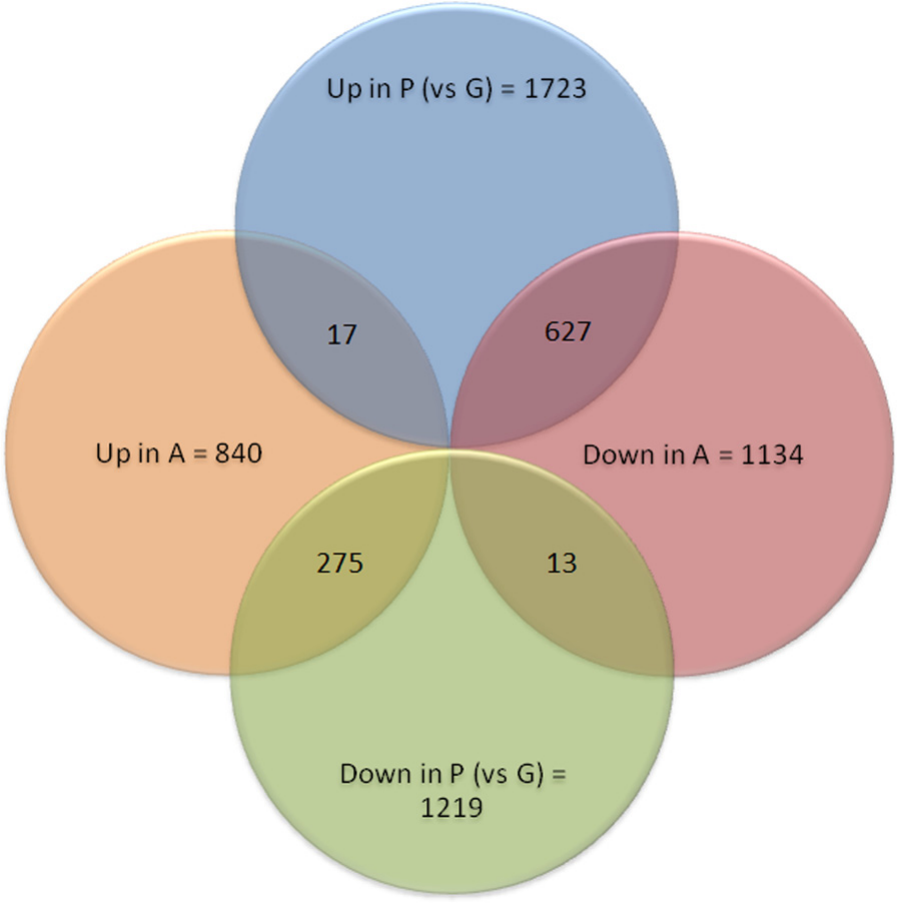
**Venn diagram of differently expressed genes.** The graph showing genes modulated according to a cut off set at 1.5 for symmetrical fold change and a p-value < 0.05. 2,942 differentially expressed genes between plateau (P) and growing (G) follicle granulosa samples. The atretic (A) versus plateau (P) contrast yielded 1974 differentially expressed genes. 932 genes modulated in both contrasts.

Normalization and a cut-off at a symmetrical fold change of >1.5 and at a p-value of <0.05 resulted in 2,942 differentially expressed genes between plateau and growing follicle granulosa samples. The atretic versus plateau contrast yielded 1974 differentially expressed based on the same parameters. 932 genes were modulated in both contrasts (Figure 3). BGA analysis showed a clear separation of sample groups, placing the plateau group as an intermediate between the growing and atretic groups, along the vertical axis (Figure 1).

**Figure 4:**
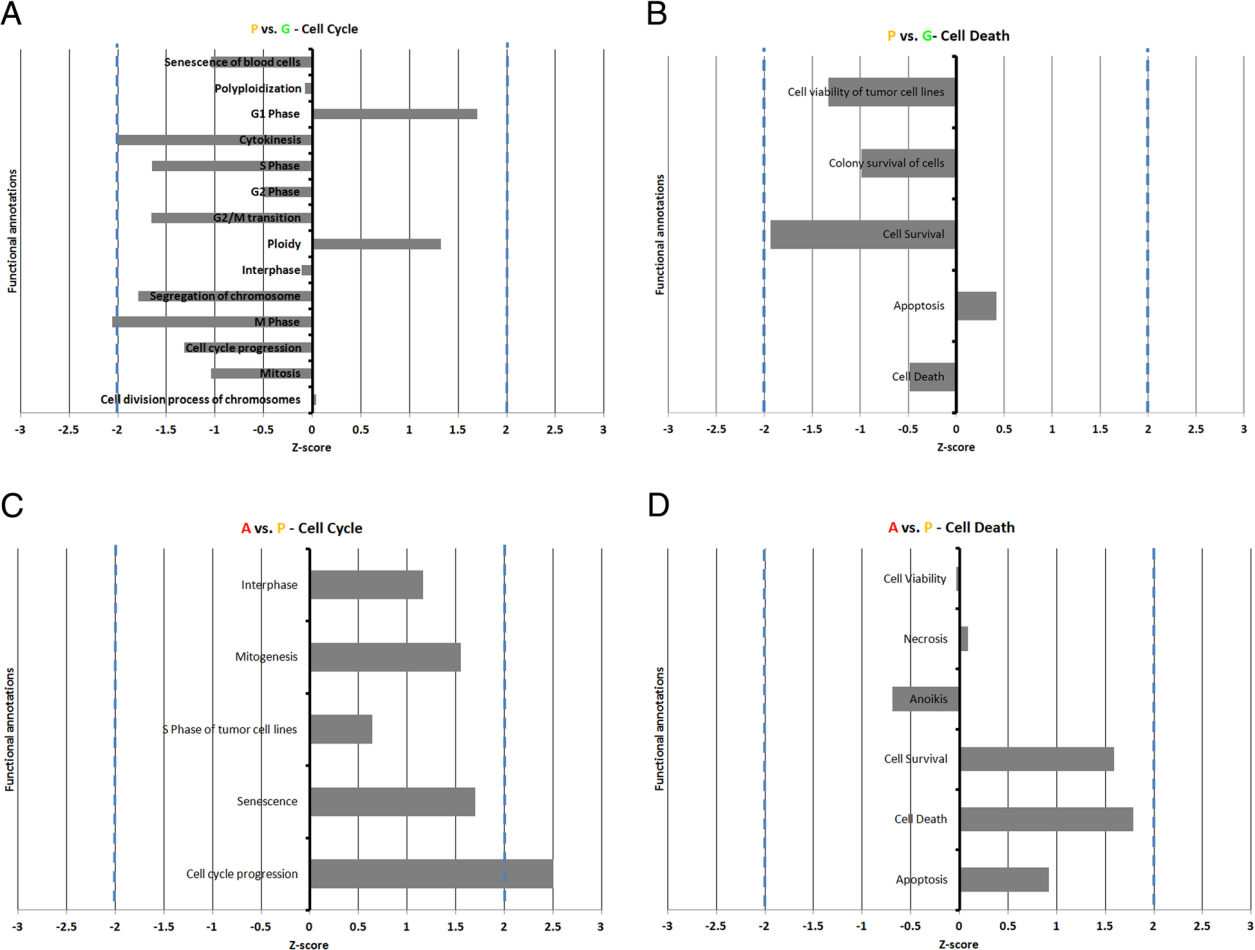
**Graphical representations of functional annotations.** The Cell Cycle **(A, C)** and Cell Death **(B, D)** functional annotations for P (plateau phase) vs. G (growing phase) and A (atretic phase) vs. P (plateau phase) are respectively presented. Dashed blue lines indicate the z-score threshold for statistical significance (-2.00 and +2.00). All annotations were above the –log(p-value) threshold indicating statistically significant overlap between genes from the study’s dataset and genes associated to the annotation in the IPA database (-log(0.05) = 1.3).

Based on the P vs. G dataset of modulated genes, IPA Functions analysis was able to make statistically significant (z-score > 2) predictions for increased activity in ploidy of cells, degradation of proteins, ploidy of fibroblasts and neuromuscular disease. The 21 functions predicted to be significantly decreased (z-score < -2) in P, relative to G, are related to cell growth, proliferation, metabolism, synthesis and energy production (Additional file 4). For differentially expressed genes in the A vs. P contrast, IPA predicted 54 functions to be increased which were related to cell movement, differentiation, cell death and, unexpectedly, cell survival and proliferation. However, no functions had a decreasing trend that was statistically significant (Additional file 4).Level-1 categories cell cycle and cell death were further analyzed as they are relevant to the physiological context of the samples. It was observed that for the P vs. G contrast, genes in the dataset had significant overlap with 14 cell cycle annotations, 11 of them showed a functional decreasing trend, most related to cell division with M-phase predicted to be significantly down-regulated in P follicles relative to G (Figure 4A). G1 Phase, cell division process of chromosomes and ploidy showed increasing trend, although not statistically significant. Five cell death annotations had significant overlap with the P vs. G dataset, with cell death, cell survival, colony survival of cells and cell viability of tumor cell lines having a decreasing trend and Apoptosis showing an increasing trend, albeit none of them were statistically significant (Figure 4B). For the A vs. P contrast, five cell cycle level 2 annotations had a significant overlap; all showing an increasing trend, with cell cycle progression predicted to be significant (Figure 4C). Six cell death annotation had a significant overlap with the A vs. P modulated genes, with cell death, apoptosis, necrosis and cell survival predicted to have an increasing trend and cell viability and anoikis predicted to be decreasing (Figure 4D). None of the A vs. P cell death annotations was statistically significant suggesting continuity between the plateau and early atretic phases.

To elucidate the presence of cell cycle progression function in the activated functions of A follicles relative to P, we analyzed the overlap of the 49 genes whose modulation indicated that cell cycle progression was activated (Figure 4C) with the genes whose modulation indicated that cell movement, cell differentiation (Additional file 4) and cell death (Figure 4D), respectively, were activated. We observed that 13 of the 49 genes involved were exclusive to the growth pattern while 36 were associated to the three other functions, including 26 genes whose modulation indicated that both cell death and cell cycle progression were increased.

As far as transcription factors are concerned, the most significant shift has been observed between growth and plateau. The growth arrest and DNA-damage-inducible, alpha (*GADD45A*) was linked to 9 of the transcription factors predicted to be activated by IPA according to the modulation of gene expression between those two phases, the highest number of connections than any other genes in the dataset (results not shown). Along with the functional annotations analysis, this represents a second observation supporting a form of continuum between the plateau phase and atresia in contrast to the growing phase.

**Figure 5:**
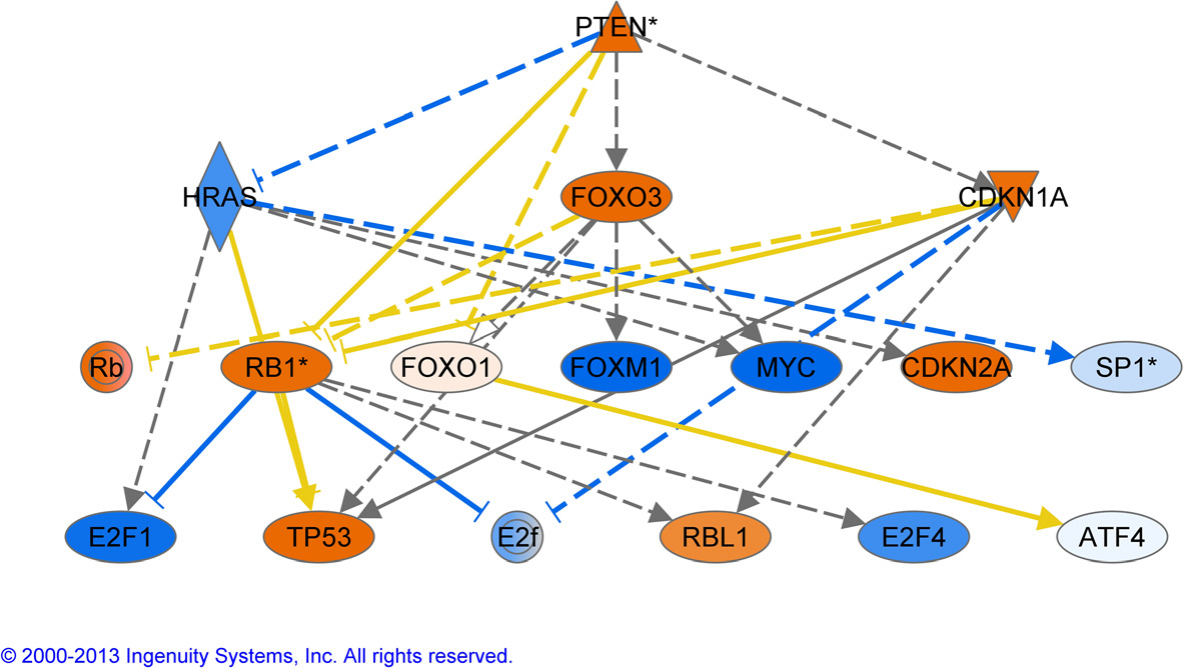
**Upstream regulator analysis in the contrast G vs P.** The regulators in orange are activated and in blue are considered as inhibited. This analysis is based on the genes that are downstream of the regulators and generates P values that were highly significant for the top regulator *PTEN. PTEN*, Phosphatase and tensin homolog ; *HRAS*, V-Ha-ras Harvey rat sarcoma viral oncogene homolog; *FOXO3*, Forkhead box O3; *CDKN1A*, Cyclin-dependent kinase inhibitor 1A; *RB1*, Retinoblastoma 1; *FOXO1*, Forkhead box O1; *FOXM1*, Forkhead box M1; *MYC*, V-myc myelocytomatosis viral oncogene homolog; *CDKN2A*, Cyclin-dependent kinase inhibitor; *SP1*, Sp1 transcription factor; *E2F1-4*, E2F transcription factor 1–4; *TP53*, Tumor protein p53; *RBL1*, Retinoblastoma-like 1 (p107); *ATF4*, Activating transcription factor 4.

**Figure 6:**
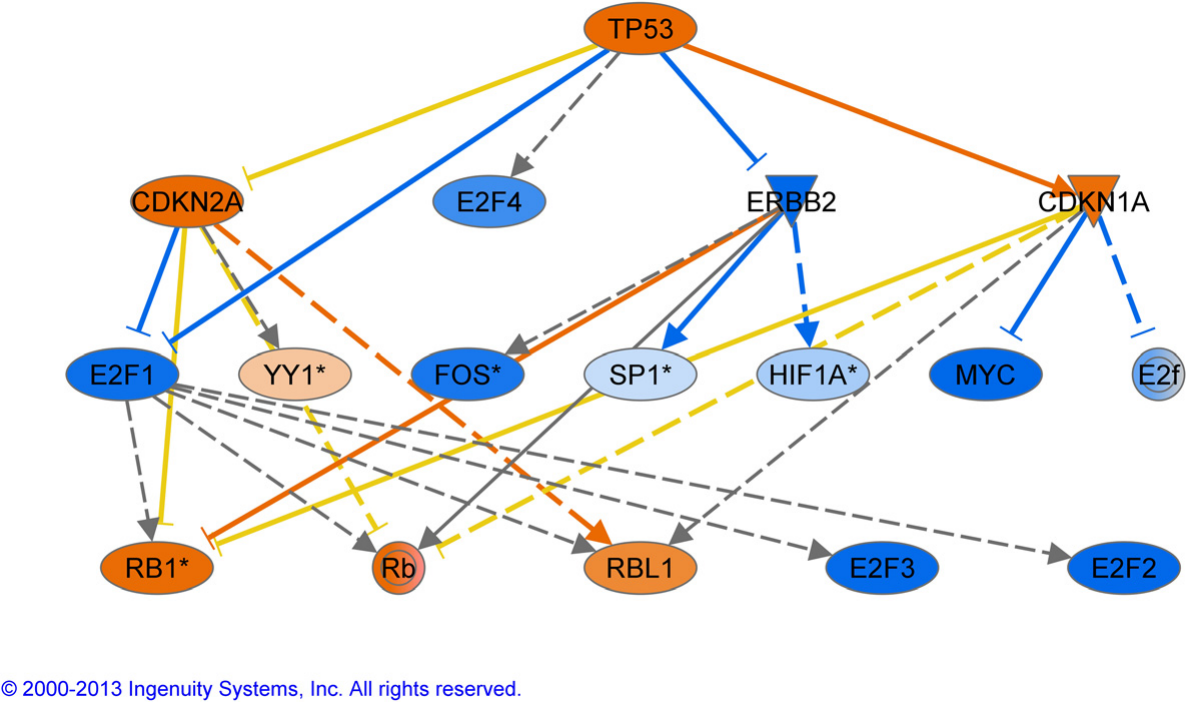
**Upstream regulator analysis in the contrast G vs P.** The regulators in orange are activated and in blue are considered as inhibited. This analysis is based on the genes that are downstream of the regulators and generates P values that were highly significant for the top regulator *TP53. TP53*, Tumor protein p53; *CDKN2A*, Cyclin-dependent kinase inhibitor; *E2F1-2-3-4*, E2F transcription factor 1-2-3-4; *CDKN1A*, Cyclin-dependent kinase inhibitor 1A; *YY1*, YY1 transcription factor; *FOS*, FBJ murine osteosarcoma viral oncogene homolog; *SP1*, Sp1 transcription factor; *HIF1A*, Hypoxia inducible factor 1, alpha subunit (basic helix-loop-helix transcription factor); *MYC*, V-myc myelocytomatosis viral oncogene homolog; *RB1*, Retinoblastoma 1; *RBL1*, Retinoblastoma-like 1 (p107).

To further illustrate the components involved when the growth becomes reduce, we have generated 2 network of upstream regulators (Figures 5 and 6) in which the impact of *PTEN* and *TP53* are described.

### Real-time PCR

**Figure 7:**
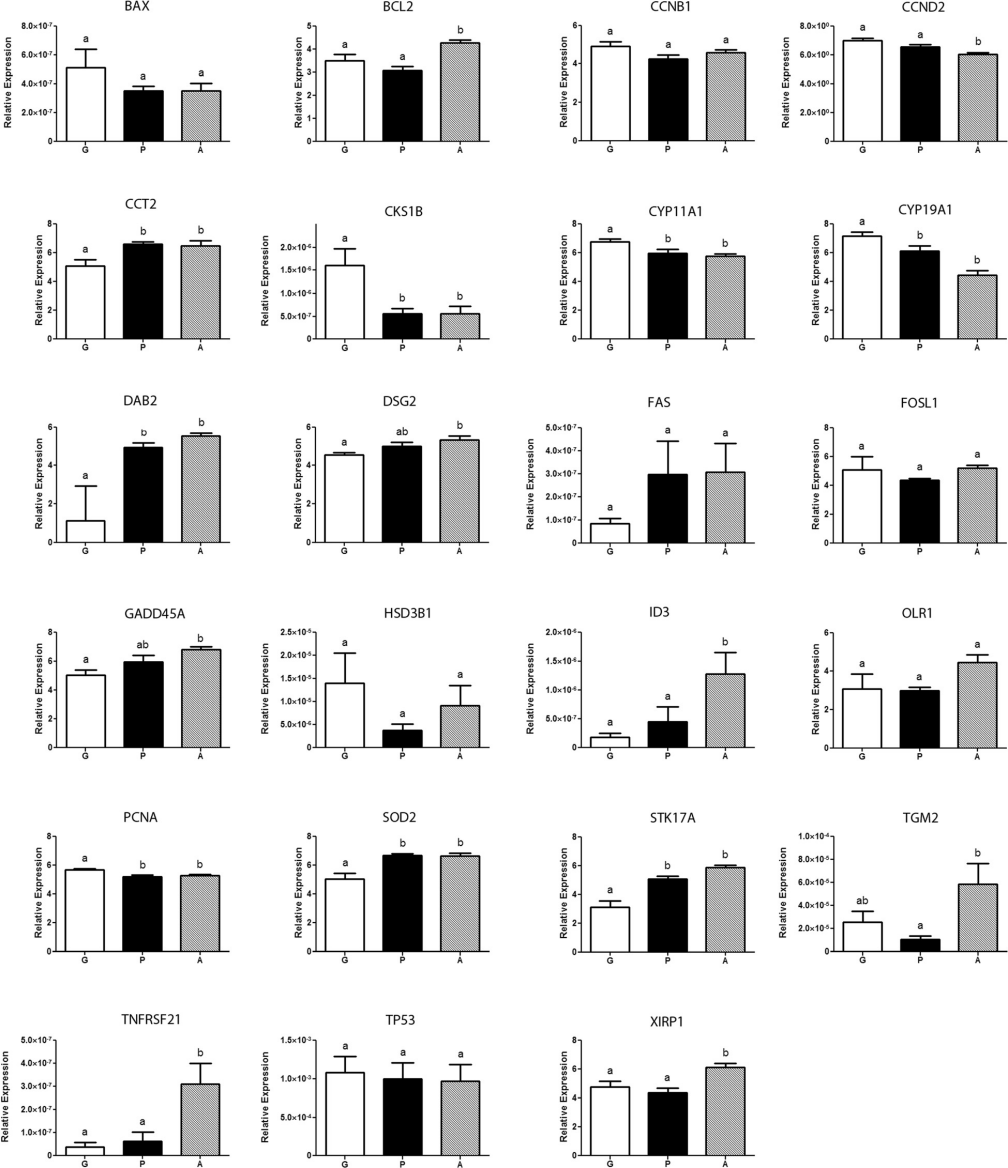
**Graphs of gene expression profiles.** 23 genes were measured by qRT-PCR in the growing (G) (white box), plateau (P) (black box) and atretic (A) (dashed box) phases (n = 7, 6 and 7 follicles). Different letters above bars correspond to significantly different expression levels (P < 0.05). Error bars represent the standard error of the mean (SEM). *BAX*, BCL2-associated X protein; *BCL2*, B-cell CLL/lymphoma 2; *CCNB1*, Cyclin B1; *CCND2*, Cyclin D2; *CCT2*, Chaperonin containing TCP1, subunit 2 (beta); *CKS1B*, CDC28 protein kinase regulatory subunit 1B; *CYP11A1*, Cytochrome P450, family 11, subfamily A, polypeptide 1; *CYP19A1*, Cytochrome P450, family 19, subfamily A, polypeptide 1; *DAB2*, Disabled-2 DSG2 Desmoglein-2; *FAS*, Fas (TNF receptor superfamily, member 6); *FOSL1*, FOS-like antigen 1; *GADD45A*, Growth arrest and DNA-damage-inducible, alpha; *HSD3B1*, Hydroxy-delta-5-steroid dehydrogenase, 3 beta- and steroid delta-isomerase 1; *ID3*, Inhibitor of DNA binding 3, dominant negative helix-loop-helix protein; *OLR1*, Low density lipoprotein (lectin-like) receptor 1; *PCNA*, Proliferating cell nuclear antigen; *SOD2*, superoxide dismutase 2, mitochondrial; *STK17A*, serine/threonine kinase 17a; *TGM2*, Transglutaminase 2 (C polypeptide, protein-glutamine-gamma-glutamyltransferase); *TNFRSF21*, Tumor necrosis factor receptor superfamily, member 21; *TP53*, Tumor protein p53; *XIRP1*, Xin actin-binding repeat containing 1.

A total of 23 genes were selected for quantification by qRT-PCR: *BAX, BCL2, CCNB1, CCND2, CCT2, CKS1B, CYP11A1, CYP19A1, DAB2, DSG2, FAS, FOSL1, GADD45A, HSD3B1, ID3, OLR1, PCNA, SOD2, STK17A, TGM2, TNFRSF21, TP53* and *XIRP1* (see Additional file 3 for gene information). The expression pattern of 11 genes: *BAX, BCL2, CKS1B, CYP11A1, CYP19A1, FAS, ID3, SOD2, TNFRSF21, TP53* and *XIRP1* were in accordance with microarray contrasts, whereby significant (P < 0.05) or non-significant (P > 0.05) differences measured on the microarray by comparing the 4 P samples against G or A respectively, were also found in the qRT-PCR results using the 4 hybridized and 2 or 3 additional samples. For 4 genes: *CCNB1, FOSL1, HSD3B1* and *OLR1*, significant differences measured by microarray, were not significant according to qRT-PCR. For 1 gene: *CCND2*, the pattern of lower mRNA expression in GC of more atretic follicles was also observed in qRT-PCR measurements, however it was observed in P vs. G only rather than in both P vs. G and A vs. P. The same was true for another gene: *PCNA*, whereby lower mRNA in P relative to G was confirmed by qRT-PCR, however it was the pattern higher mRNA levels in A relative to P that was not confirmed. For 6 genes: *CCT2, DAB2, DSG2, GADD45A, STK17A* and *TGM2*, the pattern of higher mRNA expression in GC of more atretic follicles was also observed in qRT-PCR measurements, however it was either observed in P vs. G rather than in A vs. P and/or a significant difference was observed in A vs. G. See Figure 7 for bar graphs of relative gene expression.

For *BCL2, CCNB1, CCND2, CCT2, CYP11A1, CYP19A1, DSG2, FOSL1, GADD45A, OLR1, PCNA, STK17A* and *XIRP1*, the Bartlett test for equal variance applied to raw data was significant (P < 0.001), therefore transformation by the formula Y = Log (Y*1e12) was performed and improved variance homogeneity so that the same test applied to the transformed data was non-significant (P < 0.05).

## Discussion

This is the first study evaluating the transcriptomic profiles of the growing, plateau and atretic folliculogenesis phases in large mammals. It is clear that the value of this transcriptomic data relied heavily on the ability to separate the follicles in their respective phase using flow cytometry. Although it has been shown that flow cytometry is a valid method for evaluating follicular atresia [14] and distinguishing healthy follicle from atretic ones [16] it has not been used to distinguish an intermediate growth phase for further molecular analysis. Unlike previous methods [14,16] which only took the proportion of apoptotic cells under consideration, in the current study all cell cycle phases (G1, G0/G1, S, G2) were utilized to distinguish the 3 important physiological statuses: growing, plateau and atretic. The BGA analysis indicated that cytometry was indeed able to categorize the follicle samples in three distinct groups based on transcriptomic expression of all probes on the microarray. The intermediate position of the plateau phase is supporting the assumption that the process is progressive but the different group are not on the same axe/line indicating a non-linear process. Such feature is suggesting a shift in gene expression with some being shut off and other activated.

As mentioned before, the growth phase in the bovine follicles can be distinguished in three segments; the FSH-independent growth until a follicular diameter of 2-3 mm is reached, the FSH dependant growth from 3-9 mm and the LH dependant phase from 9 mm to ovulation. Since the GC samples in this study originate from the 6-9 mm range, they can either represent follicles that are still growing, in a plateau phase (reduced FSH support) or in the atresia process (insufficient FSH) excluding dominant follicles which are larger than 8.5 mm. The first contrast analysed, P vs. G, corresponds to the reduction of cell division that is associated with decreasing FSH support [24]. In this comparison, follicles with a high proportion of mitotic granulosa cells were compared to follicles with roughly equal proportion of mitotic granulosa and apoptotic granulosa, corresponding to a turning point in follicular growth. The shift from growth to atresia is not only gradual it remains reversible for up to one day (if the dominant is ablated) indicating there is a transition period [25]. The upstream analysis further supports the inhibition of growth by the down-regulation of *MYC*, *FOS* and *E2F1-2-3* in response to *P-53* and *CDKN1A*. This later gene has been recently associated with estrogen inactive dominant follicles [26]. Using IPA, an analysis of FSH-responsive genes was performed in order to confirm that the growing follicles selected by cytometry had an expression pattern corresponding to gonadotrophin-stimulated growth. Transcripts for genes related to steroidogenesis: Cytochrome P450, subfamily XIX (*CYP19A1*, aromatase) was significantly lower in P relative to G and in A relative to P, and Cytochrome P450, family 11, subfamily A, polypeptide 1 (*CYP11A1*), was significantly lower in P relative to G. Both showed a significant decreasing linear trend from G to A. These two genes were found to be up-regulated in cultured granulosa cells when stimulated with FSH [27], demonstrating that the change in expression profile observed between growing and plateau follicles, at least for *CYP11A1* and *CYP19A1*, could be associated with FSH deprivation. Mani et al., 2010 [28] found *CYP11A1*, *CYP19A1*, but also hydroxy-delta-5-steroid dehydrogenase, 3 beta- and steroid delta-isomerase 1 (*HSD3B1*) to be up-regulated in cultured bovine GC receiving IGF1, another factor driving GC proliferation and differentiation, however the significantly lower *HSD3B1* mRNA levels in P relative to G according to microarray, were not consistent with qRT-PCR results. This suggests that the role of *HSD3B1* in granulosa may only come later in folliculogenesis, namely in the formation of the corpus luteum and the production of progesterone [29]. In addition, *HSD3B1* gene expression up-regulation could indeed be induced by FSH alone in rat granulosa [30], but not in bovine [28,31]. The aromatase cytochrome P450 enzyme, encoded by the *CYP19A1* gene is the final rate-limiting step in the synthesis of estrogens from androgens in granulosa cells. In gonadotrophin-responsive follicle, FSH enhances expression of aromatase which consequently enhances estradiol production from granulosa cells which is associated to the expression of survival genes, inhibition of apoptosis genes and promotion of follicular growth [32]. Using in situ hybridization in a recruited cohort ≥4 mm, it was observed that *CYP19A1* mRNA was significantly lower in atretic follicles compared to healthy follicles [33]. *CYP19A1* is the only gene in this study showing an expression pattern significantly different between each phase, which confirms it as an accurate marker, closely correlated to follicular health.

Some well-established granulosa cell proliferation markers also showed the expected pattern of expression across the three follicular stages, providing additional support to the precise categorization by cytometry. In mice ovaries, Cyclin D2 (*CCND2*) mRNA is expressed in proliferating granulosa, as determined by proliferating cell nuclear antigen (*PCNA*) co-localization [34], a marker for GC proliferation [35]. The mRNA and protein levels for both genes are increased in response to FSH [34,36]. This is in accordance with the current study, as *CCND2* transcript levels were found to be significantly lower in A follicles relative to G and P follicles, while *PCNA* was more highly expressed in G follicles relative to both P and A follicles. In addition, both genes showed significant decreasing trends in their expression levels when comparing each successive phase. These result suggest that although both these genes are indeed good indicators of GC proliferation, higher *PCNA* expression is characteristic of actively growing follicles, while *CCND2* expression will only go down significantly in GC once the follicle has entered advanced atresia.

Focusing on the cell cycle category, it is clear as illustrated by the 21 annotations for which there was a significant association with genes in the G vs. P dataset that it is a focal point of change in GC as the follicle enters the plateau phase. There is a clear trend towards the down-regulation of later phases of the cell cycle such as S-, G2- and M-phase, as well as cytokinesis, while the resting G1 phase is showing an increasing trend. Looking at the contrasting cell death category of functions, there is a considerably smaller overlap with the genes in the P vs. G dataset, as only 5 categories had a significant association. This indicates that the plateau phase of follicular growth, at the GC level, takes place through a negative modulation of the cell cycle machinery, more so than by the immediate activation of apoptotic or cell death pathways. This is represented in the functional analysis only by a slight positive trend of apoptosis and the broad cell death annotation not yet trending positively. The down regulation of cell survival annotations is also interesting to note as FSH mostly supports growth and proliferation by promoting downstream survival signals such as insulin like growth factors (IGF), depending on the growth stage [37]. Without those signals GC inevitably undergo apoptosis.

Among the genes contributing to the prediction that the proliferation of cells function was decreased in P vs. G, was *CKS1* and 2CDC28 protein kinase regulatory subunit 1B (*CKS1B*; also known as *CKS1*). A study investigating the role of *CKS1B* using siRNA, showed that depleting *CKS1B* in mouse embryonic fibroblasts (MEF) cells resulted in the cessation of cell proliferation [38]. *CKS1B* mRNA was significantly lower in the GC of P and A follicles relative to G follicles and there was a significant decreasing trend from G to A. Its depletion could therefore be an early indicator of GC proliferation slowing in P follicles and it remains low in A follicles where GC proliferation has stopped entirely.

As discussed previously, while the growing phase of 6-9 mm follicles is characterized by ample FSH support and their plateau phase results from a reduced supply of FSH, the atretic phase, however, is the beginning of the irreversible follicle demise which is characterised by the up regulation of specific genes. One of the genes being upregulated is *ID3. ID3* is more expressed in A relative to P and relative to G, and also showed a significant increasing trend from G to A. In mural granulosa cultured *in vitro* with FSH and/or COC, *ID3*, expression was decreased by FSH but increased by COC [39]. *ID3* acts as a dominant negative of basic helix loop helix (bHLH) transcription factor, therefore FSH and COC may regulate granulosa cell function by tuning activity of bHLH factors through *ID3*. This indicates that follicles categorized as atretic likely came from an environment low in FSH, allowing for higher expression of *ID3*, compared to follicles with high or higher FSH levels such as G and P respectively, where *ID3* expression was inhibited.

Tissue transglutaminase (*TGM2*; transglutaminase 2 (C polypeptide, protein-glutamine-gamma-glutamyltransferase)) mRNA, which is significantly up regulated in A relative to P was shown to be expressed at different levels for varying degrees of atresia in mice follicles, characterized by hematoxylin-eosin and TUNEL, suggesting it is a good indicator of the degree of follicular atresia [40]. *TGM2* is expressed in all organs and is the most ubiquitous of all the transglutaminases [41]. It has also been well documented in other tissues that *TGM2* mRNA coincides with apoptosis *in vivo*, such as the liver [42]. *TGM2* is believed to sensitize cells to apoptosis by hyperpolarizing mitochondria, an event which precedes the loss of transmembrane potential, a decrease in *GSH* levels and consequently an increase in the production of reactive oxygen species (ROS) [43]. The indication that *TGM2* transcript levels increase with the degree of atresia makes it an interesting marker for the A vs. P contrast, as both follicle type will contain apoptotic granulosa, albeit at different stages, which can explain why classical granulosa apoptosis markers such as Fas (TNF receptor superfamily, member 6; *FAS*) [44] and BCL2-associated X protein (*BAX*) [45,46] reviewed in [47] are not significantly different in their expression between the two conditions. However, B-cell CLL/lymphoma 2 (*BCL2*), a marker for apoptosis resistance [46,48], was higher in atretic follicle GC than in both P and G follicles which is unexpected [48] but not the first time such results are obtained. Valdez et al., 2005 [49] who worked with dominant follicles collected at different days of the first follicular wave found that although the number of non-viable cells increased from days 4, to days 6 and 8, there was only a significant increase in the ratio *BCL2:BAX* from day 4 to 6, with no significant difference between day 4 and 8. It can therefore be suggested that the translocation of *BAX* from the cytoplasm to the mitochondria and its interaction with *BCL2*[50] takes precedence over expression of those factors in the activation of apoptosis in bovine GC. More studies looking at protein localization will be needed to elucidate this concept. It is important to note that previous studies did not necessarily evaluate the presence of those markers in contexts matching the current study. For example, [44] found *FAS* mRNA levels to be higher in GC of the two largest, atretic subordinates than in the healthy dominant follicle. In small- and medium-sized follicle, although in an *in vitro* culture, it was found that neither *BAX* nor *BCL2* were modulated by FSH or Insulin-like growth factor 1 (*IGF1*) [28]. Since FSH is thought to modulate the dynamics of the different phases in the current study, this corresponds to the steady *BAX* levels between the three growth phases, and indicates that the increase expression of *BCL2* in A is likely driven by some other factor.

Tumor necrosis factor receptor superfamily, member 21 (*TNFRSF21*, also known as death receptor 6*: DR6*), much like other members of the TNFR family, has been shown to induce apoptosis when over expressed. In hen ovaries it was found that both transcript and protein levels were higher in cells of atretic follicles relative to healthy follicles [51]. In the current study, transcript levels were significantly up-regulated in A follicles compared to P, presenting *TNFRS21* as a marker of late follicular atresia in GC. In addition, initial experiments following *TNFRSF21* discovery showed that ectopic expression of this death receptor induced apoptosis in HeLa S3 cervical carcinoma cells [52]. As the *Functions* analysis of IPA shows, the image is not precise when comparing two tissues which are both atretic, but differing in the stage of atresia they have reached. Being among the qRT-PCR-validated genes shown to be significantly higher in A follicle GC relative to P follicle GC, *TNFRSF21* can now suggested as an indicator of advanced follicular atresia.

Desmoglein-2 (*DSG2*) [53] and chaperonin containing TCP1, subunit 2 (beta) (*CCT2*) [54] and disabled-2 (*DAB2*) [55] were significantly up-regulated in A and P relative to G. Although these genes have not been studied in granulosa specifically, it was shown in other cell types that they promote apoptosis since siRNA’s directed against their transcripts lead to a decrease in cell death. *STK17A* has been shown to induce apoptosis in multiple cell lines [56,57]. It seems to act in early atresia as his expression levels were higher in both P and A relative to G. Overexpression of FOS-like antigen 1 (*FOSL1* or fra-1) in cultured cell lines has been shown to increased apoptosis [58].

It is now clear that cell death related genes operate through many different pathways. The regular apoptosis in granulosa cells must occur with minimal inflammation and other processes seem to be activated; such as autophagy. Oxidized low density lipoprotein (lectin-like) receptor 1 (*OLR1*) mRNA expression levels were measured in the current study in order to evaluate the potential action of autophagy in the later stages of atresia, as was previously observed in quail granulosa cells [59]. Up-regulation of *OLR1* expression was associated to non-apoptotic, autophagic cell death, as determined by vacuoles and actin remodelling [60]. *OLR1* expression, although evaluated as significantly higher in A than P follicle GC by microarray data, was not observed to be significantly different between phases by qRT-PCR. However, qRT-PCR results did confirm the significant up-regulation of xin actin-binding repeat containing 1 (*XIRP1*), a gene involved in actin cytoskeleton re-organization [61] and the most up-regulated gene in A compared to P in microarray data, may also indicate that autophagy is more prominent in late atretic follicle than in early atretic follicles. This could correspond to a greater proportion of the granulosa cell population having entered apoptosis and consequently a greater quantity of cellular debris needing to be digested.

Transcript levels for *BCL2, ID3, TGM2, TNFRSF21* and *XIRP1* were all significantly higher in A follicles GC relative to P and G, indicating that they are indicators of late atresia. *CCT2* and *STK17A* were significantly higher in both P and A relative to G, and could therefore be used to differentiate between growing and early-atretic follicles. The same can be proposed for *CKS1B, CYP11A1* and *CYP19A1*, for which the levels seem to decrease significantly when the follicle passes from growing phase to the plateau phase and remain at lower levels throughout atresia, except for *CYP19A1* whose transcription decreases even more. Finally, *DSG2* and *GADD45A* were significantly higher in A follicles compared to G follicles, with P follicles showing an intermediate levels, which resulted in a significant increasing trend from G to A.

The difference in functional activity between the P vs. G contrast and the A vs. P contrast, as predicted by IPA software, is striking. While 21 functional annotations were predicted to be decreased in P relative to G and only were predicted to 4 increased, no functions were predicted to be decreased in A relative to P but no less than 54 were predicted to be increased. This is a strong suggestion that the plateau or static phase really corresponds to a quiescent or resting stage, intermediate between the active, FSH-supported growth phase and the atretic process.

The functions predicted to be increased, in a statistically significant way, in the A follicle GC relative to P follicle GC revolve around tumorigenesis, cell cycle progression and survival of cells as well as apoptosis and cell death, development of various cell types, differentiation of various cell type, homeostasis, proliferation and multiple cell movement related functions. For example, the functional annotation with the highest z-score is cell movement of neutrophils. Neutrophils are polymorphonuclear leukocytes, or white blood cells, and have been shown to be recruited to the thecal compartment of pre-ovulatory follicles just before ovulation [62], in corpus lutea (CL) and increase during CL regression, suggesting a role in both ovulation and luteolysis [63]. These cells are also present in higher numbers among thecal cells in atretic follicles relative to healthy follicles [64]. The results of the current study complement these results by specifying that infiltration and movement of leukocytes is a hallmark specific to the advanced atretic follicles. It also suggests that granulosa are likely the source of signals attracting these white blood cells to the surrounding thecal compartment and that atresia may indeed be a differentiation process similar to ovulation and later luteolysis.

Based on microarray results, *GADD45A* was modulated and linked to 9 of the predicted activated transcription factors in the P vs. G contrast. However, qRT-PCR validation showed that relative expression was only significantly higher in A follicles GC, relative to G, with P at an intermediate level, resulting in a significant increasing trend from G to A. It is therefore possible that these transcription factors drive the expression of *GADD45A* mRNA as early as the plateau phase, but this only result in a significant difference in transcript levels when the atretic stage is reached. This qualifies *GADD45A* as another indicator of late-atresia. *GADD45a* has been shown to cause cell cycle arrest at the G2-M transition by binding Cdc2 within the Cdc2/cyclin B1, a complex required to complete the G2-M transition, causing the dissociation of the complex and preventing cell cycle progression [65]. It has also been shown that transfection of a Gadd45a expression vector has induced apoptosis, reportedly by interacting *MEKK4/MTK1* and activating the *JNK/p38* signalling which induces apoptosis [66]. Although they extracted RNA from both the thecal cells (TC) and granulosa cells (GC) at once, from bovine follicles, [67] found significantly higher levels of *GADD45A* transcripts in the second largest follicle of the first follicular wave (7.8 ± 0.2 mm) than in the largest one (10.7 ± 0.7 mm). In addition, they found, using in situ hybridization, that *GADD45A* mRNA was expressed in both TC and GC in atretic follicles but only in GC in healthy follicles, as classified by progesterone and estradiol concentration in follicular fluid. They suggest that these results indicate that *GADD45A* activity is increasingly needed for the progression of apoptotic cell death in follicular atresia and stress the fact that stage-specific gene expression may closely reflect the follicle’s growth or atresia. All of the seven transcription factors predicted to be activated by IPA and reported to drive *GADD45A* expression in the literature: breast cancer 1, early onset (*BRCA1*), forkhead box O3 (*FOXO3* or *FKHRL1*), forkhead box O4 (*FOXO4* or *AFX1*), myogenic differentiation 1 (*MYOD1*), tumor protein p53 (*TP53*) (see also Figures 5– 6), tumor protein p63 (*TP63*) and tumor protein p73 (*TP73*) have been shown to be involved with apoptosis [68-74].

Very recently, two large scale genomic analysis were published by Rodgers (12, 13) where small follicles are compared to large ones and healthy follciles from less than 5 mm compared to atretic ones. For the second study, closer to the work reported here but in a different follicle class (smaller than 5 mm compared to us 6-9 mm), the focus is on the extracellular matrix and demonstrate how important the matrix is for the survival and growth of the follicle. Only one gene was validated with PCR, the aromatase (*CYP19A1*) and as in our results, decreases significantly in atretic follicles. For their upstream regulators (our Figure 5 and their table eight) several pathways are common between these 2 follicles classes: P53 is activated and MYC is inhibited. These are complementary datasets that will lead to a better understanding of the folliculogenesis process.

IPA also revealed significant overlap between differently-expressed genes (DEG) of both contrasts and the NRF2-mediated oxidative stress response canonical pathway, which leads to the activation of the transcription factor NRF2 (*NFE2L2*, nuclear factor (erythroid-derived 2)-like 2). This transcription factor responds to environment insult [75] including reactive oxygen species (ROS) [76]. It was observed that ROS generated by the mitochondria play an important role in the release of cytochrome *c* and other molecules which lead to the activation of apoptosis [77]. Indeed, in granulosa, FSH acts to suppress ROS, generated by steroidogenesis and metabolic activities, within the granulosa by stimulating the synthesis of the antioxidant glutathione (*GSH*) [78]. In the same study, it was shown that blocking *GSH* synthesis lead to GC apoptosis. Multiple target genes of *NFE2L2* were modulated in both contrast based on microarray data. Among them, *SOD2* expression is upregulated by *NFE2L2* according to the IPA database. Its protein encodes the mitochondrial isoform of the superoxide dismutases. It removes the superoxide anion in the dismutation reaction producing hydrogen peroxide and molecular oxygen. *SOD2* transcript levels were greater in P and A follicles relative to G. This may indicate that withdrawal of FSH leads to an increased ROS production, which in turn leads to the activation of the *NFE2L2* oxidative response pathway and the expression of *SOD2* antioxidant enzyme to prevent runaway ROS accumulation as part of the atretic process. Although more target genes would have needed to be validated by qRT-PCR to confirm the intermediate role of *NFE2L2*, this results still indicates a role for increased *SOD2* transcription in follicular atresia. More validation with PCR and protein measurements are now required to dissect each of these specific pathways but strong hypothesis can now be put forward to better explain the follicular dynamics.

## Conclusions

This study confirmed cytometry as a valid method to differentiate between apoptotic GC originating from atretic follicles and growing follicles, but went further by showing that the method could also be successful in differentiating between the early-atretic, plateau phase and late atresia. This allowed the identification of genes whose expression in GC could be used to differentiate between the statuses of the follicle. The novel Functions component of IPA software’s core analysis allowed the integration of large microarray datasets and provided valuable insight as to the status of key cellular function. It indicated that as the follicle passes from an actively growing phase to the plateau phase, growth is slowed mostly by halting the cell division machinery in GC, not yet activating cell death mechanisms, and engaging into a quiescent phase as very few functions were predicted to be increased. However, as the follicle advances further into atresia, GC become more proactive in the activation of cell death. These results put forth the initial structuring of gene pathways implicated in folliculogenesis and a first step towards a complete folliculome.

## Competing interests

The authors declare that they have no competing interests.

## Authors’ contributions

GD and MS conceived the study and wrote the paper. GD performed the experiments and analysis. Both authors read and approved the final manuscript.

## Supplementary Material

Additional file 1 List of differentially expressed genes between Plateau and Growing phases. Click here for file

Additional file 2 List of differentially expressed genes between Atretic and Plateau phases. Click here for file

Additional file 3 qRT-PCR primer sequences, product size, annealing temperature and accession number for the studied genes. Click here for file

Additional file 4 Functions analysis in Ingenuity Pathway Analysis (IPA) software for the P vs. G (A) and A vs. P (B) contrasts. Click here for file
