# Supplementary material for: Changes in granulosa cells gene expression associated with growth, plateau and atretic phases in medium bovine follicles
Source: J Ovarian Res. 2014 May 7;7:50. doi: 10.1186/1757-2215-7-50 (PMC4046060; doi:10.1186/1757-2215-7-50)
Supplement: Additional file 3 — qRT-PCR primer sequences, product size, annealing temperature and accession number for the studied genes. [file 1757-2215-7-50-S3.doc]

**Additional file 3: qRT-PCR primer sequences, product size, annealing temperature and accession number for the studied genes.**

| **Gene Symbol** | **Gene Description** | **Primer sequence** | | **Product Size (bp)** | **Annealing Temperature (°C)** | **Accession Number** |
| --- | --- | --- | --- | --- | --- | --- |
| BAX | BCL2-associated X protein | fwd: | CAGCTCTGAGCAGATCATGAAGAC | 396 | 57 | NM_173894.1 |
|  |  | rev: | TCTCGAAGGAAGTCCAATGTCCAG |  |  |  |
| BCL2 | B-cell CLL/lymphoma 2 | fwd: | TGTGGATGACCGAGTACCTGAA | 232 | 57 | NM_001166486.1 |
|  |  | rev: | CTTCTGCATATTTGTTGGCGGCAG |  |  |  |
| CCNB1 | Cyclin B1 | fwd: | GAGGAAGAGCAAGCAGTCAAAC | 277 | 55 | NM_001045872.1 |
|  |  | rev: | CAGTCACAAAGGCAAAGTCACC |  |  |  |
| CCND2 | Cyclin D2 | fwd: | GATCTCCTGGCAAAGATCACCAAC | 247 | 57 | NM_001076372.1 |
|  |  | rev: | TACAACCAGAGAGAAGGAGAGAGC |  |  |  |
| CCT2 | Chaperonin containing TCP1, subunit 2 (beta) | fwd: | CCAGTAGAACACCAGGCAAAGAAG | 374 | 57 | NM_001034239.1 |
|  |  | rev: | CTATGAACTGGTCCAGAGCTCAAC |  |  |  |
| CKS1B | CDC28 protein kinase regulatory subunit 1B | fwd: | GCAGAGTGATCATGTCGCACAAAC | 163 | 57 | NM_001113311.1 |
|  |  | rev: | CTCTGCTGAACACCAAGATTCCTC |  |  |  |
| CYP11A1 | Cytochrome P450, family 11, subfamily A, polypeptide 1 | fwd: | ATCCAGTGTCTCAGGACTTCGT | 209 | 57 | NM_176644.2 |
|  |  | rev: | GAACATCTTGTAGACGGCATCA |  |  |  |
| CYP19A1 | Cytochrome P450, family 19, subfamily A, polypeptide 1 | fwd: | GCGTTGTCTAAGCAAACTCTCCCA | 462 | 60 | NM_174305.1 |
|  |  | rev: | CAGCTTCCAACTGGCATTTCCCAT |  |  |  |
| DAB2 | Disabled-2 | fwd: | GGCTTCTCTTCAACCCACATCATC | 310 | 57 | NM_001193246.1 |
|  |  | rev: | CTTCGCTTACATTCGGCTAGTGAG |  |  |  |
| DSG2 | Desmoglein-2 | fwd: | CTAGACGCCCTTCAATCCATGT | 353 | 56 | NM_001192172.2 |
|  |  | rev: | CGGTAGGCACTTGAGGATATACAG |  |  |  |
| FAS | Fas (TNF receptor superfamily, member 6) | fwd: | CTTGATGTGCATCCTGGTAGACTG | 256 | 57 | NM_174662.2 |
|  |  | rev: | CACCAACAGAGAACACATGGCAAG |  |  |  |
| FOSL1 | FOS-like antigen 1 | fwd: | CCCTATGGTCTTCATCACCTCTTC | 353 | 56 | NM_001205985.1 |
|  |  | rev: | CAAATGGTCTCCCTTTAGCCTCTG |  |  |  |
| GADD45A | Growth arrest and DNA-damage-inducible, alpha | fwd: | GGGAAAGTCGCTACATGGATCAGT | 232 | 57 | NM_001034247.1 |
|  |  | rev: | CCATCTGCAAAGTCATCTATCGCC |  |  |  |
| HSD3B1 | Hydroxy-delta-5-steroid dehydrogenase, 3 beta- and steroid delta-isomerase 1 | fwd: | GGATGAGCCTTCCTATTTCTCTGC | 317 | 57 | NM_174343.2 |
|  |  | rev: | CTGCTAACTAATGTCCACGTTCCC |  |  |  |
| ID3 | Inhibitor of DNA binding 3, dominant negative helix-loop-helix protein | fwd: | CTTGTGATCTCCAACGACCAAAGG | 328 | 57 | NM_001014950.1 |
|  |  | rev: | GTACACCTCCACACACTGAAAGAC |  |  |  |
| OLR1 | Low density lipoprotein (lectin-like) receptor 1 | fwd: | ACTTCACCGCCAGAACCTGAATCT | 295 | 60 | NM_174132.2 |
|  |  | rev: | ACCATCTTCCCAAAGCCACGAGTA |  |  |  |
| PCNA | Proliferating cell nuclear antigen | fwd: | GGACAGTGCCTTCATTTGGACTAC | 330 | 57 | NM_001034494.1 |
|  |  | rev: | AAGACTCCTGTACAAGGCTCCCTA |  |  |  |
| SOD2 | superoxide dismutase 2, mitochondrial | fwd: | GAGAAGGGTGATGTTACAGCTCAG | 479 | 57 | NM_201527.2 |
|  |  | rev: | CTTGGTGTACTCAGTGTAAGGCTG |  |  |  |
| STK17A | serine/threonine kinase 17a | fwd: | CAGATACTCAGAAGCCAGAAACCG | 387 | 57 | NM_001083422.1 |
|  |  | rev: | TAACTTCACCCGTTAAGCCTCCTC |  |  |  |
| TGM2 | Transglutaminase 2 (C polypeptide, protein-glutamine-gamma-glutamyltransferase) | fwd: | AGTTGCCTATGCTGATCACCCA | 430 | 58 | NM_177507.2 |
|  |  | rev: | TCAGTGCTCGCTGTTTGTAGCT |  |  |  |
| TNFRSF21 | Tumor necrosis factor receptor superfamily, member 21 | fwd: | CCTCACAAGCCATCACGACTTTAC | 349 | 57 | NM_001076911.1 |
|  |  | rev: | GCACGTCTATAAATGGGAGCAGAG |  |  |  |
| TP53 | Tumor protein p53 | fwd: | TCGGGAGAGGTCAGAATGTGTTCC | 409 | 60 | NM_174201.2 |
|  |  | rev: | CTTTGGCACTGAGGTTCACCAAGG |  |  |  |
| XIRP1 | Xin actin-binding repeat containing 1 | fwd: | CAGAAACACACGCTTCTCACACAG | 308 | 56 | NM_001245928.1 |
|  |  | rev: | CTAGGGACATTGGTGATCGTAGAC |  |  |  |
| ACTB | actin, beta | fwd: | ATCGTCCACCGCAAATGCTTCT | 101 | 59 | NM_173979.3 |
|  |  | rev: | GCCATGCCAATCTCATCTCGTT |  |  |  |
| B2M | beta-2-microglobulin | fwd: | AGACACCCACCAGAAGATGG | 234 | 54 | NM_173893.3 |
|  |  | rev: | GGGGTTGTTCCAAAGTAACG |  |  |  |
| GAPDH | glyceraldehyde-3-phosphate dehydrogenase | fwd: | CCAACGTGTCTGTTGTGGATCTGA | 217 | 58 | NM_001034034.2 |
|  |  | rev: | GAGCTTGACAAAGTGGTCGTTGAG |  |  |  |
