# Supplementary material for: Changes in granulosa cells gene expression associated with growth, plateau and atretic phases in medium bovine follicles
Source: J Ovarian Res. 2014 May 7;7:50. doi: 10.1186/1757-2215-7-50 (PMC4046060; doi:10.1186/1757-2215-7-50)
Supplement: Additional file 4 — Functions analysis in Ingenuity Pathway Analysis (IPA) software for the P vs. G (A) and A vs. P (B) contrasts. [file 1757-2215-7-50-S4.doc]

**Additional File 4.** **Functions analysis in Ingenuity Pathway Analysis (IPA) software for the P vs. G (A) and A vs. P (B) contrasts.** Only functions annotations that obtained a Regulation z-score value higher than the absolute value of 2, which is considered significant and therefore for which IPA could predict the Activation State, are presented. P-value < 0.05 calculated by Fisher’s Exact test, # Molecules: number of molecules associated to annotation in user’s dataset.

1. **P vs. G - Statistically significant function trends**

|  | | | | | |
| --- | --- | --- | --- | --- | --- |
| **Category** | **Functions Annotation** | **p-Value** | **Predicted Activation State** | **Regulation z-score** | **# Molecules** |
| Cell Cycle | ploidy of cells | 3.69E-06 | Increased | 2.253 | 22 |
| Protein Synthesis | degradation of protein | 1.17E-05 | Increased | 2.166 | 71 |
| Cell Cycle | ploidy of fibroblasts | 6.35E-05 | Increased | 2.079 | 4 |
| Neurological Disease | neuromuscular disease | 6.90E-06 | Increased | 2.003 | 196 |
| Cellular Growth and Proliferation | growth of tumor cell lines | 4.02E-03 | Decreased | -2.013 | 103 |
| Post-Translational Modification | ubiquitination of protein | 9.70E-04 | Decreased | -2.018 | 36 |
| Cell Cycle | entry into mitosis of cervical cancer cell lines | 5.54E-03 | Decreased | -2.031 | 4 |
| Cell Cycle | M phase | 6.45E-11 | Decreased | -2.054 | 49 |
| Cellular Assembly and Organization | chromosomal congression of chromosomes | 5.24E-04 | Decreased | -2.097 | 5 |
| Reproductive System Disease | tumorigenesis of genital tumor | 5.59E-04 | Decreased | -2.098 | 10 |
| Cell Cycle | M phase of tumor cell lines | 3.11E-06 | Decreased | -2.138 | 20 |
| Cancer | tumorigenesis | 2.67E-13 | Decreased | -2.18 | 531 |
| Cell Cycle | cycling of centrosome | 1.71E-04 | Decreased | -2.282 | 13 |
| Reproductive System Disease | genital tumor | 4.93E-05 | Decreased | -2.309 | 135 |
| Developmental Disorder | congenital anomaly of brain | 3.84E-03 | Decreased | -2.353 | 13 |
| Nucleic Acid Metabolism | metabolism of nucleotide | 4.99E-03 | Decreased | -2.391 | 59 |
| Nucleic Acid Metabolism | metabolism of nucleoside triphosphate | 2.25E-05 | Decreased | -2.458 | 28 |
| Nucleic Acid Metabolism | biosynthesis of purine nucleotide | 5.53E-05 | Decreased | -2.462 | 26 |
| Nucleic Acid Metabolism | biosynthesis of nucleoside triphosphate | 4.61E-04 | Decreased | -2.463 | 14 |
| Energy Production | synthesis of ATP | 3.38E-03 | Decreased | -2.468 | 11 |
| Cellular Growth and Proliferation | proliferation of cells | 1.36E-10 | Decreased | -2.656 | 350 |
| Nucleic Acid Metabolism | biosynthesis of purine ribonucleotide | 2.56E-05 | Decreased | -2.67 | 17 |
| Cellular Growth and Proliferation | growth of cells | 5.02E-06 | Decreased | -2.682 | 241 |
| DNA Replication, Recombination, and Repair | orientation of chromosomes | 1.07E-08 | Decreased | -3.062 | 13 |
| DNA Replication, Recombination, and Repair | alignment of chromosomes | 6.86E-08 | Decreased | -3.062 | 12 |
|  |  |  |  |  |  |
| © 2000-2012 Ingenuity Systems, Inc. All rights reserved. | |  |  |  |  |

1. **A vs. P - Statistically significant function trends**

|  | | | | | |
| --- | --- | --- | --- | --- | --- |
| **Category** | **Functions Annotation** | **p-Value** | **Predicted Activation State** | **Regulation z-score** | **# Molecules** |
| Cellular Movement | cell movement of neutrophils | 4.63E-03 | Increased | 3.288 | 30 |
| Cellular Growth and Proliferation | proliferation of tumor cells | 3.67E-05 | Increased | 3.239 | 31 |
| Cancer | digestive organ tumor | 2.40E-07 | Increased | 3.208 | 154 |
| Cancer | carcinoma | 1.77E-14 | Increased | 3.105 | 305 |
| Cellular Movement | infiltration by neutrophils | 2.60E-03 | Increased | 3.046 | 17 |
| Cellular Growth and Proliferation | proliferation of cancer cells | 1.58E-04 | Increased | 2.995 | 23 |
| Cellular Movement | cell movement of granulocytes | 2.88E-03 | Increased | 2.995 | 38 |
| Cellular Movement | migration of tumor cell lines | 1.13E-05 | Increased | 2.994 | 57 |
| Cellular Development | differentiation of blood cells | 4.91E-04 | Increased | 2.986 | 73 |
| Cellular Movement | migration of cells | 1.85E-07 | Increased | 2.976 | 168 |
| Cellular Development | differentiation | 4.33E-06 | Increased | 2.956 | 187 |
| Cellular Movement | infiltration of granulocytes | 4.25E-03 | Increased | 2.858 | 21 |
| Cellular Development | differentiation of lymphocytes | 5.82E-04 | Increased | 2.844 | 51 |
| Cellular Movement | cell movement of myeloid cells | 7.42E-04 | Increased | 2.804 | 53 |
| Tissue Development | neuritogenesis | 1.12E-03 | Increased | 2.794 | 43 |
| Cellular Development | differentiation of mononuclear leukocytes | 5.13E-04 | Increased | 2.733 | 55 |
| Cellular Development | differentiation of cells | 6.21E-06 | Increased | 2.725 | 176 |
| Cancer | solid tumor | 1.10E-13 | Increased | 2.72 | 307 |
| Cellular Development | differentiation of leukocytes | 8.33E-04 | Increased | 2.704 | 64 |
| Cellular Development | developmental process of lymphocytes | 2.41E-03 | Increased | 2.522 | 63 |
| Cell Cycle | cell cycle progression | 1.59E-04 | Increased | 2.507 | 101 |
| Tissue Development | morphogenesis of neurites | 6.84E-05 | Increased | 2.498 | 21 |
| Cellular Movement | cell movement | 1.55E-08 | Increased | 2.488 | 190 |
| Cellular Movement | cell movement of phagocytes | 1.53E-03 | Increased | 2.485 | 52 |
| Organismal Injury and Abnormalities | damage of mice | 5.69E-03 | Increased | 2.461 | 9 |
| Cancer | metastasis | 1.64E-10 | Increased | 2.456 | 71 |
| Hematological System Development and Function | hematopoiesis | 7.85E-05 | Increased | 2.451 | 92 |
| Cellular Function and Maintenance | homeostasis of leukocytes | 1.10E-03 | Increased | 2.394 | 53 |
| Cellular Function and Maintenance | Lymphocyte homeostasis | 1.47E-03 | Increased | 2.394 | 52 |
| Cellular Movement | infiltration of myeloid cells | 4.26E-03 | Increased | 2.326 | 23 |
| Cellular Development | development of mononuclear leukocytes | 4.81E-04 | Increased | 2.323 | 56 |
| Cancer | tumorigenesis of malignant tumor | 4.22E-03 | Increased | 2.321 | 30 |
| Cellular Movement | homing of cells | 6.54E-03 | Increased | 2.273 | 49 |
| Cellular Development | developmental process of T lymphocytes | 2.92E-03 | Increased | 2.241 | 51 |
| Cellular Growth and Proliferation | proliferation of tumor cell lines | 3.04E-06 | Increased | 2.235 | 98 |
| Cell Death | apoptosis of endothelial cells | 6.65E-03 | Increased | 2.218 | 16 |
| Cellular Movement | cell movement of tumor cell lines | 6.52E-05 | Increased | 2.205 | 68 |
| Cardiovascular Disease | vascular disease | 2.72E-03 | Increased | 2.17 | 60 |
| Cancer | tumorigenesis of carcinoma | 1.59E-03 | Increased | 2.166 | 23 |
| Cellular Development | development of lymphocytes | 6.78E-04 | Increased | 2.146 | 55 |
| Cell Death | cell death of cervical cancer cell lines | 1.20E-03 | Increased | 2.131 | 32 |
| Cancer | endocrine gland tumor | 7.51E-05 | Increased | 2.117 | 47 |
| Cellular Development | development of leukocytes | 2.38E-04 | Increased | 2.082 | 60 |
| Cellular Function and Maintenance | T cell homeostasis | 3.99E-03 | Increased | 2.075 | 48 |
| Cellular Development | T cell development | 4.13E-03 | Increased | 2.074 | 47 |
| Cellular Development | differentiation of T lymphocytes | 1.12E-03 | Increased | 2.067 | 38 |
| Cellular Development | development of blood cells | 5.07E-04 | Increased | 2.046 | 65 |
| Tissue Development | morphogenesis of dendrites | 1.67E-03 | Increased | 2.045 | 8 |
| Tissue Development | development of dendrites | 4.80E-03 | Increased | 2.045 | 12 |
| Cellular Growth and Proliferation | proliferation of cells | 1.34E-06 | Increased | 2.041 | 251 |
| Cancer | cancer | 5.52E-17 | Increased | 2.035 | 374 |
| Cellular Development | differentiation of multilineage progenitor cells | 3.13E-03 | Increased | 2.029 | 5 |
| Cell Death | survival of tumor cell lines | 3.16E-04 | Increased | 2.02 | 51 |
| Tissue Morphology | size of lesion | 1.28E-03 | Increased | 2.006 | 26 |
|  |  |  |  |  |  |
| © 2000-2012 Ingenuity Systems, Inc. All rights reserved. | |  |  |  |  |
